# Viral antigens detectable in CSF exosomes from patients with retrovirus associated neurologic disease: functional role of exosomes

**DOI:** 10.1186/s40169-018-0204-7

**Published:** 2018-08-27

**Authors:** Monique R. Anderson, Michelle L. Pleet, Yoshimi Enose-Akahata, James Erickson, Maria Chiara Monaco, Yao Akpamagbo, Ashley Velluci, Yuetsu Tanaka, Shila Azodi, Ben Lepene, Jennifer Jones, Fatah Kashanchi, Steven Jacobson

**Affiliations:** 1 Department of Pathology, University of Virginia School of Medicine, Charlottesville, VA 22901 USA; 2 Viral Immunology Section, Neuroimmunology Branch, National Institute for Neurological Disease and Stroke, National Institutes of Health, 10 Center Drive Rm 5C103, Bethesda, MD 20892 USA; 3 Laboratory of Molecular Virology, George Mason University, Manassas, VA 20110 USA; 4 Laboratory of Molecular Medicine and Neuroscience, National Institutes for Neurological Disease and Stroke, National Institutes of Health, Bethesda, MD 20892 USA; 5 Department of Immunology, University of the Ryukyus Graduate School of Medicine, Okinawa, 903-0125 Japan; 6 Ceres Nanosciences, Manassas, VA 20109 USA; 7 Vaccine Branch, National Cancer Institute, National Institutes of Health, Bethesda, MD 20892 USA

**Keywords:** Exosomes, Nanotraps, Spontaneous proliferation, Specific lysis, HTLV-1 Tax, Cell-free virus

## Abstract

**Background:**

HTLV-1 infects over 20 million people worldwide and causes a progressive neuroinflammatory disorder in a subset of infected individuals called HTLV-1 associated myelopathy/tropical spastic paraparesis (HAM/TSP). The detection of HTLV-1 specific T cells in the cerebrospinal fluid (CSF) suggests this disease is immunopathologically mediated and that it may be driven by viral antigens. Exosomes are microvesicles originating from the endosomal compartment that are shed into the extracellular space by various cell types. It is now understood that several viruses take advantage of this mode of intercellular communication for packaging of viral components as well. We sought to understand if this is the case in HTLV-1 infection, and specifically if HTLV-1 proteins can be found in the CSF of HAM/TSP patients where we know free virus is absent, and furthermore, if exosomes containing HTLV-1 Tax have functional consequences.

**Results:**

Exosomes that were positive for HTLV-1 Tax by Western blot were isolated from HAM/TSP patient PBMCs (25/36) in ex vivo cultures by trapping exosomes from culture supernatants. HTLV-1 seronegative PBMCs did not have exosomes with Tax (0/12), (Fisher exact test, *p *= 0.0001). We were able to observe HAM/TSP patient CSF (12/20) containing Tax^+^ exosomes but not in HTLV-1 seronegative MS donors (0/5), despite the absence of viral detection in the CSF supernatant (Fisher exact test *p *= 0.0391). Furthermore, exosomes cultivated from HAM/TSP PBMCs were capable of sensitizing target cells for HTLV-1 specific CTL lysis.

**Conclusion:**

Cumulatively, these results show that there are HTLV-1 proteins present in exosomes found in virus-free CSF. HAM/TSP PBMCs, particularly CD4^+^CD25^+^ T cells, can excrete these exosomes containing HTLV-1 Tax and may be a source of the exosomes found in patient CSF. Importantly, these exosomes are capable of sensitizing an HTLV-1 specific immune response, suggesting that they may play a role in the immunopathology observed in HAM/TSP. Given the infiltration of HTLV-1 Tax-specific CTLs into the CNS of HAM/TSP patients, it is likely that exosomes may also contribute to the continuous activation and inflammation observed in HAM/TSP, and may suggest future targeted therapies in this disorder.

**Electronic supplementary material:**

The online version of this article (10.1186/s40169-018-0204-7) contains supplementary material, which is available to authorized users.

## Background

Human T cell lymphotropic virus type 1 (HTLV-1) is a human delta retrovirus that targets the immune system, primarily infecting CD4^+^ T cells [1, 2]. Infection for most individuals results in little to no clinical sequelae, although ~ 1–5% of patients will develop a chronic, progressive myelopathy termed HTLV-1 associated myelopathy/tropical spastic paraparesis (HAM/TSP) [3]. HAM/TSP patients typically present with bladder/bowel dysfunction, lower limb spasticity, ataxia and paresthesias after decades of infection [4]. Patient immune cells are characterized by continuous proliferation, activation, and lack of immune suppression [5, 6]. While immunologic studies have demonstrated a chronic infiltration of activated CD4^+^ and CD8^+^ T cells into the central nervous system (CNS) with disease progression [7, 8], HTLV-1 has not been shown to actively infect neurons, oligodendrocytes, or microglia in vivo [9, 10].

Similar to other viruses that establish persistent infections, HTLV-1 is adept at replicating while evading the immune system; it tightly controls the expression of viral genes in cells to escape potential immune recognition [11, 12]. However, there are multiple reports indicating detection of viral proteins extracellularly [13–15]. Indeed, while neither the provirus nor the viral transcript has been demonstrated in non-cell associated material such as serum and cerebrospinal fluid (CSF) [16], HTLV-1 specific T cells have been found within HAM/TSP CSF, parenchyma and spinal cord [17, 18]. Intact extracellular viral antigens suggest that viral proteins may be exported by viral co-opting of the exosomal pathway.

Exosomes are unilamellar extracellular vesicles that originate from the endosomal compartment in all cells [19]. Exosomes begin as intraluminal vesicles (ILVs) which aggregate to form multivesicular bodies (MVBs) as they progress through the endosomal sorting complex required for transport (ESCRT) pathway [20, 21]. Ultimately, once the MVBs fuse with the plasma membrane in an alternate pathway to lysosomal degradation, ILVs are released to the extracellular space where they are subsequently termed exosomes [22, 23]. In appearance, exosomes are cup-shaped vesicles that range in size from 30 to 100 nm [24, 25], although the definition has recently expanded to include all vesicles less than 150 nm [26], as they all originate from the ESCRT/endosomal compartment [27, 28]. These vesicles have been shown to shuttle intracellular proteins, mRNAs, and miRNAs to neighboring and distal sites in a recently discovered form of intracellular communication [29]. Within the immune system, exosomes have been shown to play a role in T cell activation via dendritic cell (DC) presentation of reprocessed internalized exosomal antigens [30, 31] or capture and presentation of exosomes at the cell surface [21, 32]. Alternatively, T cells produce exosomes that can educate DCs [33]. In particular, regulatory T cells (Tregs) which are a type of T cell important in immune homeostasis and suppression of activated immune responses, are a prolific source of exosomes in the T cell compartment [34]. Tregs produce exosomes containing miRNAs which have been shown to aid in suppression of activated T cells as well as reduce the ability of DCs to activate T cells [34, 35]. CD4^+^CD25^+^ T cells, including Tregs, also are the primary reservoir of HTLV-1 in infected individuals [6, 36]. As Treg dysfunction has been implicated in HAM/TSP [5, 37, 38], assessment of exosomes from the regulatory T cells subset becomes important.

In addition to CD4^+^CD25^+^ T cells, HTLV-1 is capable of infecting several other cell types of the immune system, including dendritic cells, CD8^+^ T cells, and monocytes [1, 39–41]. As exosomes have been demonstrated to play a vital role in the intercellular communication that occurs in the immune system [21, 42], an understanding of how virus-infected immune cells change exosomal function is important. Several human viruses have been shown to incorporate viral components into exosomes. Herpesviruses, flaviviruses, bunyaviruses, and lentiviruses have all demonstrated incorporation of viral proteins and miRNAs into exosomes or alteration of host contents of immune cell derived exosomes [43–46]. HTLV-1 also appears to employ this mechanism in vitro [47], however it is unclear what, if any, role incorporation of viral components into exosomes might play ex vivo in the pathogenesis of HTLV-I associated diseases. In addition to their role in the immune system, exosomes have also been shown to cross the blood–brain barrier (BBB) into the CNS and into other immune privileged sites [48, 49]. In patients, exosomes from the periphery have been implicated as progenitors of neuroinflammation, therefore it is essential to elucidate if exosomes can be found in the CNS of HAM/TSP patients and if these exosomes are similarly neuroinflammatory [50].

Here we demonstrated that Nanotrap^®^ particle-isolated exosomes from HAM/TSP patient PBMCs contained HTLV-1 Tax protein. Importantly, HTLV-1 Tax protein was found in exosomes directly isolated from HAM/TSP patient cerebrospinal fluid (CSF) supernatant previously found to be negative for free HTLV-1 virus, indicating the presence of HTLV-1 viral antigen in the non-cellular fraction. Furthermore, HAM/TSP exosomes could sensitize uninfected target cells for lysis by HTLV-1 specific cytotoxic T lymphocytes (CTLs). Collectively, the incorporation of HTLV-1 products into exosomes may represent a mechanism by which viral antigens could be transported to the CNS and be targeted by virus-specific immune responses associated with the immunopathogenesis of HAM/TSP. In addition, the detection of exosomes containing viral antigen may be a biomarker of viral exposure particularly in sites (i.e., CSF) that are absent of cell-free virus.

## Results

### Nanotrapping exosomes from tissue culture supernatants

**Fig. 1:**
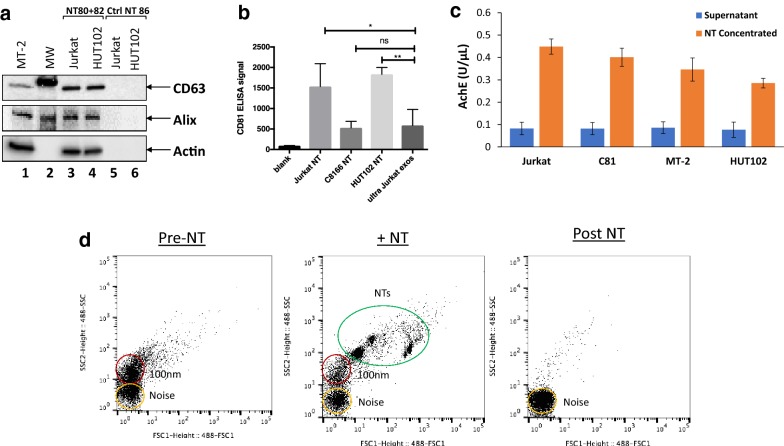
Nanotrapping exosomes from tissue culture supernatants. **a** CD63, Alix, and Actin levels from exosomes nanotrapped by either NT80 + 82 particles (lanes 3 and 4) or by NT86 particles (Ctrl NT 86; lanes 5 and 6) from Jurkat (HTLV-1 uninfected) and HUT102 (HTLV-1 infected) tissue culture supernatants were analyzed by Western blot. MT-2 whole cell extracts and molecular weight (MW) ladder are shown (lanes 1 and 2). **b** CD81 ELISA signals were measured from isolated exosomes as a measure of exosome concentration. Exosomes were nanotrapped (designated by NT) from HTLV-1 negative Jurkat, and HTLV-1 infected C8166 and HUT102 cells from 1 mL of starting tissue culture supernatant. The background is designated as blank. Ultracentrifuged Jurkat exosomes (ultra Jurkat exos) were also measured. Statistical analysis was performed by unpaired *t*-test. *ns* not significant; *p-value < 0.05; **p-value < 0.01 (n − 4). **c** Measurement of acetylcholinesterase (AchE) activity associated with exosome populations was performed. Tissue culture supernatant was measured for AchE activity from Jurkat, C8166 (C81), MT-2, and HUT102 cells both prior to nanotrapping (blue) and after addition of NT80 + 82 and subsequent exosome isolation (orange). **d** Tissue culture supernatants from HTLV-1 infected cells were analyzed by specialized flow cytometry for NanoFACS. Left panel represents a representative sample prior to addition of Nanotrap^®^ particle (Pre-NT), middle panel represents sample with addition of Nanotrap^®^ particle (+NT) and right panel represents sample after Nanotrap^®^ particle and exosomes have been removed (Post NT). NTs are shown circled in green, while 100 nm vesicles are circled in red, and noise is circled in yellow

Previously, the utility of Nanotrap^®^ (NT) particles (NT80) has been shown in capturing viral protein-containing exosomes from HTLV-1 infected cell line supernatants, while other particles were shown to be more specific for viral capture (NT86) [47, 51]. However, more recent applications of NTs have successfully used the combination of two particles (NT80 + 82) for the concentration of exosomes from extracellular milieu [51, 52]. Therefore, to test the efficacy of this combination with HTLV-1 exosomes, we initially isolated exosomes from tissue culture supernatants from HUT102 (HTLV-1 infected) and Jurkat (HTLV-1 uninfected) cell lines using Nanotrap^®^ technology. NT80 + 82 particles pulled down exosomes from both cell lines, regardless of HTLV-1 infection status, as demonstrated by the presence of Alix, CD63, and Actin (Fig. 1a, lanes 3 and 4). Control NT particles, NT86 (known to isolate viruses but not exosomes [53]), was unable to isolate exosomes from tissue culture supernatant, as shown by a lack of Alix, CD63, or Actin (Fig. 1a, lanes 5 and 6).

Next, exosomes nanotrapped from several T cell lines were assessed for CD81, another exosomal marker. As shown in Fig. 1b, using an electrochemical based ELISA, CD81 signals for exosomes isolated by NT particles from 1 mL of tissue culture media from Jurkat, C8166, and HUT102 cells was equal to or greater than the CD81 signal detected from ultracentrifuged Jurkat exosomes from 10 mL of starting material (Fig. 1b). These data signify that Nanotrap^®^ particles can be used to concentrate exosomes to equal or greater levels than those isolated from 10 times the same starting material by conventional ultracentrifugation.

To further characterize the efficiency of Nanotrap^®^ particles in exosomal capture, samples were assessed for acetylcholinesterase (AchE) activity before and after NT80 + 82 concentration [54, 55]. Prior to NT isolation, exosomes were present at low levels in tissue culture supernatant of Jurkat, C81 (C8166), MT-2, and HUT102 cells, as demonstrated by low levels of AchE activity (Fig. 1c, blue bars). However, after nanotrapping with NT80 + 82, there was a fourfold increase in AchE activity consistent with an increase in exosome concentration (Fig. 1c, orange bars).

Samples were additionally analyzed by nanoFACS, a technique which quantifies vesicles by forward and side scatter (FSC, SSC) [56]. As seen in Fig. 1d, there was a population of vesicles approximately 100 nm in size in tissue culture supernatants before nanotrapping (left panel). After the addition of NT particles to the supernatant, these NT particles could be visualized by a shift in FSC vs. SSC, indicating an increase in size (Fig. 1d, middle panel). Moreover, the decrease in the 100 nm population was visualized after the addition of the NT particles, signifying extraction of the exosomes by the NTs (Fig. 1d, middle panel). After trapping, the 100 nm vesicles (exosomes) were removed from the tissue culture supernatant, leaving the post-NT sample absent of this population (Fig. 1d, right panel). In addition, exosome visualization was performed by laser capture of the Brownian motion of extracellular vesicles with Nanosight (Additional file 1: Figure S1). Pre-nanotrapped samples showed a population of 2 × 10^9^ 109 nm vesicles along with 5 × 10^8^ 311, 477 and 583 nm vesicles (left panel). NTs alone showed a population of particles sized 215–600 nm in size, as expected (middle panel). However, the resultant nanotrapped exosomes and NTs after pulldown were denoted by peaks from 215 to 600 nm (right panel). Additionally, a population of ~ 3.5 × 10^9^ 145 nm vesicles had been pulled from the initial tissue culture supernatant. Analysis of the vesicles from pre- and post-NT supernatants showed that small (< 150 nm) vesicles were reduced by nanotrapping, while medium (200–300 nm) and large (> 300 nm) vesicles increased after nanotrapped vesicles were removed from the media. This may indicate the selective nanotrapping of small vesicles (exosomes). Collectively, these results support nanotrapping with NT80 + 82 particles as an efficient method for exosome isolation.

### HTLV-1 infected cell lines have exosomes containing HTLV-I Tax protein

**Fig. 2:**
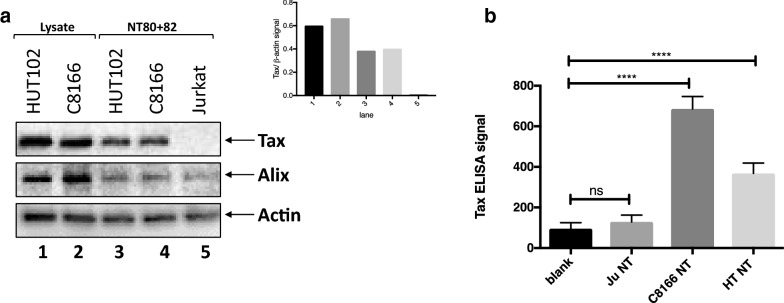
HTLV-1 infected cell lines have exosomes containing HTLV-1 Tax protein. **a** Western blots for levels of HTLV-1 Tax, Alix and β-actin (Actin) proteins were performed from cell lysates and NT80 + 82 nanotrapped exosomes from HUT102 and C8166 (C81) cells. Jurkat cell supernatants were also nanotrapped with NT80 + 82 particles and run alongside infected cell NT pulldowns. Tax levels detected were normalized to the β-actin signal for each sample and quantified in the upper right inset. **b** HTLV-1 Tax was measured by electrochemical ELISA. Nanotrapped (NT80 + 82) exosomes from Jurkat (Ju), C8166 (C81), and HUT102 (HT) cells were freeze-thawed and then measured for HTLV-1 Tax reactivity. Statistical significance was determined by unpaired *t*-test. ****p-value < 0.0001 (n = 4)

As shown in Fig. 1, all cell lines tested produced exosomes in culture, regardless of infection status. However, it has also been previously shown that viral infection can alter exosome cargo. Indeed, HTLV-1 infected cell lines were shown to produce exosomes that contained HTLV-1 Tax protein, which could be isolated by ultracentrifugation and NT80 particles [47]. Here, we used 1 mL of cell-free, filtered (0.22 μm) tissue culture supernatant to demonstrate that of the nanotrapped exosomes isolated from Jurkat, C8166, and HUT102 cells, HTLV-1 Tax protein was only detectable by Western blot from the exosomes isolated from the HTLV-1 infected cell lines C8166 and HUT102 (Fig. 2a, lanes 3 and 4). Levels of Tax were normalized to the levels of Actin to further emphasize that relatively high levels of Tax in comparison to Actin were present in infected cell exosomes, while there were undetectable levels present from uninfected cells (Fig. 2a, inset). In addition, exosomes containing HTLV-1 Tax were also confirmed using ELISA. Exosomes were freeze-thawed after nanotrapping to open the vesicles, after which only exosomes isolated from the HTLV-1 infected cell lines C8166 and HUT102 showed ELISA reactivity signal for HTLV-1 Tax above background (Fig. 2b). These data indicate that use of NT80 + 82 particles to concentrate exosomes from HTLV-1 infected cells can allow one to capture exosome-associated Tax with reliable specificity.

### Exosome production from cultured PBMCs

**Fig. 3:**
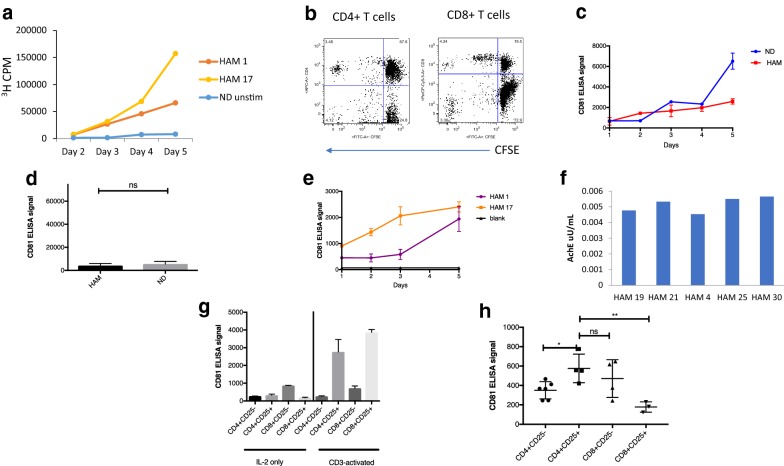
Exosome production from cultured PBMCs. **a** Normal donor (ND) or HAM/TSP (HAM) PBMCs were cultured in exosome free media and measured over 2–5 days for ^3^H-thymidine uptake in counts per minute (CPM). NDs were left unstimulated by IL-2 or CD3 (ND unstim). **b** Carboxyfluorescein succinimidyl ester (CFSE) signal by HAM/TSP PBMCs was measured after 5 days in culture. One representative graph is pictured. **c** ELISAs were performed over 5 days for CD81 from nanotrapped (NT80 + 82) exosomes from ND and HAM PBMCs. ND PBMCs were maintained with IL-2 to induce proliferation. **d** Exosomes were nanotrapped (NT80 + 82) after 5 days culture of HAM or ND PBMCs (n = 5), followed by ELISA for CD81. ND PBMCs were maintained with IL-2 to induce proliferation. **e** A CD81 ELISA was performed from nanotrapped (NT80 + 82) exosomes isolated from HAM 1 and HAM 17 patient samples over 5 days in culture. **f** Vesicles isolated from HAM PBMC (HAM 4, 19, 21, 25, and 30) short-term cultures by NT pulldown were measured for AchE activity. **g** ND PBMCs were sorted immediately after thawing (IL-2 only) or after activation for 1 day with anti-CD3 100 ng/mL and 100 IU/mL IL-2 (activated). **h** HAM/TSP PBMCs were cultured for 24 h prior to FACS sorting into CD4^+^CD25^−^, CD4^+^CD25^+^, CD8^+^CD25^−^, and CD8^+^CD25^−^ T cells (n = 4). Statistical analysis was performed by unpaired *t*-test. *p-value < 0.05; **p-value < 0.01

We next investigated peripheral blood mononuclear cells (PBMCs) as a potential source of exosomes in HAM/TSP, since HTLV-1 primarily infects immune cells [57]. Exosomes were isolated from normal donor (ND) and HAM/TSP PBMCs after a short term 5-day culture. Through a process of spontaneous lymphoproliferation, it is well known that HAM/TSP PBMCs can proliferate in culture without the addition of exogenous antigens or cytokines [58, 59]. However, ND PBMCs do not proliferate without the addition of a stimulating agent, such as IL-2. To analyze the growth of our HAM/TSP PBMCs in comparison to unstimulated ND cells over time, we performed a ^3^H-thymidine uptake assay. Maximum proliferation was seen after 5 days in culture, as demonstrated by peak ^3^H-thymidine uptake by representative HAM/TSP patients compared to an ND (Fig. 3a). Moreover, both CD4^+^ and CD8^+^ T cells from HAM/TSP patients proliferated as seen by CFSE diminution on flow cytometry (Fig. 3b). Exosome production from proliferating PBMCs was likewise analyzed. Maximum CD81 detection occurred after 5 days in culture for both HAM and IL-2 stimulated ND PBMCs (Fig. 3c). No significant difference between CD81 signals from IL-2 stimulated ND and HAM/TSP PBMCs was detected (Fig. 3d). To directly compare exosomes produced from HAM/TSP PBMCs from 2 different patients, CD81 levels were measured by ELISA over 5 days. Maximum exosome detection was once again found to be at 5 days for both patient PBMCs (Fig. 3e). Vesicles isolated from several HAM/TSP PBMC 5 days cultures by NT pulldown were further confirmed as exosomes by detection of AchE activity from samples (Fig. 3f). Water served as a negative control for which no AchE activity could be detected (data not shown). All samples showed roughly similar levels of AchE activity. From these results, 5 days was therefore chosen as the optimal time point for analysis of exosomes isolated from PBMC short-term cultures.

HAM/TSP patients are associated with an activated immune response, and here we showed that PBMCs from HAM/TSP patients produce exosomes during active proliferation (Fig. 3e, f). Given prior reports that demonstrated that exosome production increases after activation of mouse and human PBMCs [60], it was of interest to determine which T cell subset’s exosome production was most influenced by activation. Therefore, the effect of CD3 activation on T cell exosome production was first evaluated in NDs (Fig. 3g). As it has previously been shown that Treg cells (CD4^+^CD25^+^) are both prolific producers of exosomes and a primary target for HTLV-1 infection [6, 34, 36], we first focused our analysis on this subset of T cell. ND PBMCs were either activated by CD3 treatment prior to sorting into T cell subsets, or sorted first into T cell subpopulations and then maintained with IL-2 in culture. As shown in Fig. 3g, relative to IL-2 treated T cell cultures, CD3 activation increased exosome production from predominantly CD4^+^CD25^+^ and CD8^+^CD25^+^ T cell subsets, implicating Treg cells as a predominant producer of exosomes in activated PBMC T cells.

As HTLV-1 in HAM/TSP patients is known to predominantly infect and activate CD4^+^ T cells [10], we next analyzed the production of exosomes in HAM/TSP T cell subsets. Similar to exogenously CD3^−^ activated ND PBMCs, endogenously stimulated HAM/TSP CD4^+^CD25^+^ produce more exosomes than CD4^+^CD25^−^ cells (Fig. 3h). However, in contrast to activated ND PBMCs, CD8^+^CD25^−^ T cell exosomes from HAM/TSP patients produced a higher CD81 signal while CD8^+^CD25^+^ T cell exosomes produced minimal CD81 signal. This may be indicative of a different CD8^+^ T cell subset that could potentially be responsible for greater release of exosomes in comparison to the CD8^+^CD25^+^ T cells in HAM/TSP patients. Together, these results indicate that CD4^+^CD25^+^ T cells (Tregs) are likely one of the main culprits in the generation of exosomes during both T cell activation and HTLV-1 infection in HAM/TSP patients.

### Cultured HAM/TSP PBMCs produce exosomes containing HTLV-1 Tax

**Fig. 4:**
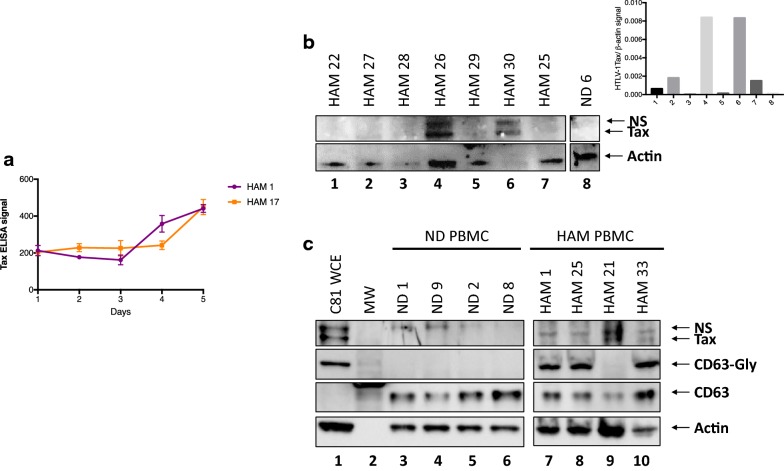
Cultured HAM/TSP PBMCs produce exosomes containing HTLV-1 Tax. **a** HAM 1 and HAM 17 PBMCs were cultured over 5 days and were assessed for HTLV-1 Tax content from isolated exosomes by electrochemical ELISA. **b** A representative Western blot of HTLV-1 Tax and Actin from nanotrapped (NT80 + 82) exosomes of 7 HAM/TSP patient PBMCs after 5 days culture. Tax compared to Actin (β-actin) levels was quantified for the Western blot and shown in the upper right insert. *NS* nonspecific band. **c** An additional Western blot of HTLV-1 Tax, CD63 (two forms: glycosylated (CD63-Gly) and unmodified), and Actin from nanotrapped (NT80 + 82) exosomes of 4 ND and 4 HAM/TSP patient PBMCs after 5 days culture was performed. ND PBMCs were cultured with 100 IU/mL IL-2 while HAM/TSP PBMCs received no exogenous cytokines. Samples were run on a 4–20% Tris–glycine gel, followed by overnight wet transfer to PVDF membranes. C81 whole cell extract (WCE) was utilized as Tax positive control. *NS* nonspecific band, *MW*  molecular weight ladder

Previously, it has been shown that HTLV-1 infected cell lines produce exosomes that contain Tax protein [47]. Likewise, we similarly showed that Tax-containing exosomes could be captured from HTLV-1 infected cell lines using NT80 + 82 particles (Fig. 2). However, to our knowledge, Tax has not been shown to be present in exosomes from primary HTLV-1 infected cells. Along these lines, we cultured HAM/TSP PBMCs from two patients for 1–5 days to determine if HTLV-1 Tax protein could similarly be detected by ELISA from the subsequent nanotrapped exosomes. As shown in Fig. 4a, cultured PBMCs from the 2 HAM/TSP patients produced exosomes containing HTLV-1 Tax that increased over this 5-day period. This trend corresponds nicely to the total exosome production (shown by CD81 levels) previously seen from the same patients’ cultured PBMCs (Fig. 3e). Tax was additionally observed in the majority of HAM patient exosomes at varying levels by Western blot (Fig. 4b, c). As expected, exosomes isolated from ND PBMCs did not contain Tax protein. Tax signal above Actin was observed in every lane except 3 and 8, representing 1 HAM/TSP patient and a ND respectively (Fig. 4b, inset). Data in Fig. 4c shows that, similar to Fig. 4b, the majority of HAM/TSP patient PBMCs contained varying levels of Tax. Interestingly, levels of glycosylated CD63 (CD63-Gly) differed strikingly between ND and HTLV-1 infected samples. Only samples infected with HTLV-1, including the C81 whole cell lysates, possessed CD63-Gly, whereas exosomes from all samples had associated unmodified CD63. Cross-sectional analysis of the HAM/TSP cohort by Western Blot demonstrated that 69.4% (25/36) were positive for HTLV-1 Tax in exosomes isolated from cultured PBMCs, while no Tax positive (Tax^+^) exosomes were produced from cultured PBMC of HTLV-1 seronegative controls (0/12) (Fisher exact test: *p*-value = 0.0001).

### Detection of exosomes containing HTLV-1 Tax in CSF of HAM/TSP patients

**Fig. 5:**
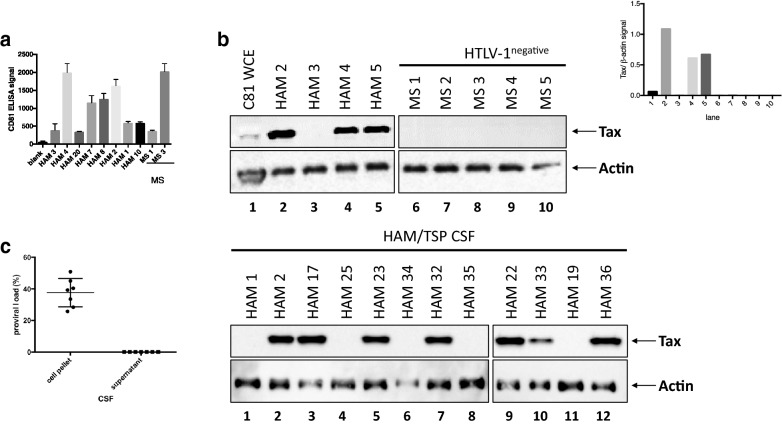
Detection of exosomes containing HTLV-1 Tax in CSF of HAM/TSP patients. **a** Cell-free CSF samples from 8 HAM/TSP patients and 2 MS patients were nanotrapped (NT80 + 82) and analyzed by ELISA for CD81 signal to determine levels of isolated exosomes. **b** Four HAM/TSP (HAM) and 5 multiple sclerosis (MS) patient CSF samples were nanotrapped (NT80 + 82), followed by Western blot analysis for Tax and Actin. C8166 whole cell extract (C81 WCE, lane 1) was added as a positive control. Tax normalized to Actin (β-actin) signal in the upper Western blot panel was quantified and is shown in the upper right inset. **c** Twelve HAM/TSP CSF samples were incubated with NT80 + 82 particles, followed by Western blot analysis for Tax and Actin levels. **d** Presence of HTLV-1 virus was assessed by analysis for the provirus. Pelleted cells and corresponding cell-free supernatants from HAM/TSP CSF samples were probed for HTLV-1 Tax by digital droplet PCR (ddPCR)

HAM/TSP is a neuropathologically mediated disorder and therefore the detection of exosomes in the CSF is of significant interest. Cell-free CSF was obtained from 4 HAM/TSP patients and 5 HTLV-1 seronegative multiple sclerosis (MS) control patients and directly trapped by NT80 + 82 to be analyzed by Western blot for the presence of HTLV-1 Tax. Measurable, but variable exosome levels were detected from both HAM/TSP and MS CSF samples as quantified by CD81 ELISA reactivity (Fig. 5a). Moreover, 3 of 4 initial HAM/TSP CSF samples had exosomes with detectable Tax compared to none of the control MS CSF samples (Fig. 5b). This was verified by assessing Tax above Actin signal for the upper Western blot panel, where signal was only detected from lanes 2, 4, and 5 (Fig. 5b, inset). An additional 12 HAM/TSP CSF samples are shown in Fig. 5c in which 7 are positive for Tax containing exosomes. It is remarkable to note the significant levels of Tax observed in some of the HAM/TSP CSF samples by Western blot. While Tax was detected at low levels from HTLV-1 infected cell line extracts (Fig. 5b, lane 1), Tax from HAM/TSP patient CSF exosomes was far more abundant, when present. However, not all HAM/TSP CSF exosomes tested were Tax^+^ as detectable by Western blot (12/20 tested, 60%). Interestingly, the detection of exosomes containing Tax from HAM/TSP CSF did not correlate with the total numbers of exosomes isolated (data not shown). As it is well established that HTLV-1 is a cell-associated virus and restricted to cells in the CSF [61], we tested the proviral load of the cell pellet vs. the supernatant of several HAM/TSP CSF samples. We confirmed that while the cell pellet showed expected levels of HTLV-1 provirus, the CSF supernatant was HTLV-1 virus-free (Fig. 5d). These data therefore show the presence of exosomes containing HTLV-1 Tax in the CSF of HAM/TSP patients in the supernatant compartment from which no HTLV-1 virus can be detected.

Since subsets of HAM/TSP patient PBMCs (Fig. 4b) and CSF samples (Fig. 5b) were shown to contain HTLV-1 Tax^+^ exosomes, we asked if there were correlations between these two compartments. All tested HAM/TSP patients (12/12) with Tax^+^ exosomes in the CSF were also positive for Tax in exosomes from cultured ex vivo PBMCs (data not shown). For CSF samples from HAM/TSP patients that were negative for Tax in the CSF, 3/8 were also negative in the exosomes isolated from ex vivo PBMCs (data not shown). These results suggest that Tax^+^ exosomes in the CSF could reflect levels of systemic infection.

### HAM/TSP exosomes can sensitize targets to antigen-specific responses

**Fig. 6:**
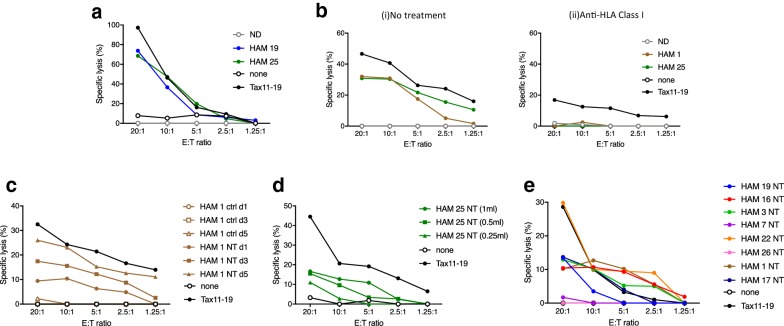
HAM/TSP exosomes can sensitize targets to Ag-specific responses. **a** Cytotoxic T lymphocyte (CTL) lysis was analyzed after the addition of HAM/TSP or ND PBMC-derived exosomes or Tax11-19 peptide. **b** HAM/TSP or ND PBMC-derived exosomes or Tax11-19 peptide pulsed targets were co-cultured with anti-HLA Class I ab (MCA81EL 5 μg/mL) and CTL target lysis was analyzed. **c** HAM/TSP PBMC supernatants were either nanotrapped with NT80 + 82 (NT) or with a control nanotrap (NT86) unable to isolate exosomes (ctrl). Supernatants collected after day 1, day 3, or day 5 of culture were used for trapping. **d** Various amounts (1 mL, 500, and 250 μL) of 5 days HAM 25 PBMC tissue culture supernatants were used as the starting material for nanotrapping by NT80 + 82 particles (NT), followed by CTL assay. **e** Eight representative HAM/TSP patient PBMC-derived exosomes were utilized for additional analysis of CTL lysis by CTL assay

As exosomes containing HTLV-1 Tax in CSF were detectable in the majority of HAM/TSP patients (Fig. 5b, c), it was of interest to determine if Tax^+^ exosomes were immunologically functional. Therefore, we investigated the potential of exosomes produced by HAM/TSP PBMCs to sensitize targets for HTLV-1 Tax-specific CTL lysis. HTLV-1 Tax is highly immunogenic and HTLV-1 Tax-specific CTLs have been detected in brain and spinal cord from HAM/TSP biopsy and autopsy cases [18, 62–64]. To determine if exosomes containing Tax could sensitize target cells for lysis by Tax-specific CTLs, autologous lymphoblasts from a HAM/TSP patient were pulsed with exosomes generated from HAM/TSP patients or NDs. A representative experiment is shown in Fig. 6a. Exosomes containing Tax from 2 HAM/TSP patient PBMCs significantly sensitized target cells for CTL lysis at levels comparable to control Tax peptide [65, 66] pulsed targets, whereas Tax^−^ exosomes from representative NDs (n = 4) did not sensitize targets for CTL-mediated lysis (Fig. 6a). Donor antigen presenting cells (APC) pulsed with Tax11-19 peptide (black circle) yielded 100% specific lysis at 20:1 E:T ratio and decreased at lower E:T ratios. HAM/TSP PBMC-derived exosomes (blue and green circle) yielded close to 80% specific lysis of donor APCs at 20:1 E:T ratio and decreased at lower E:T ratios. ND (gray open circle) did not sensitize target donor APCs for lysis at any E:T ratio. CD8^+^ antigen specific T cell lysis is known to be HLA class I restricted. As shown in Fig. 6b(i, ii), anti-HLA class I antibodies blocked lysis of control Tax11-19 peptide (black circle) and HAM/TSP exosome-pulsed targets (brown and green circles).

To demonstrate that these observations were specific for exosomes containing Tax from HAM/TSP patients, control nanotraps (ctrl NT86) were used and failed to isolate exosomes that could sensitize targets for CTL lysis (Fig. 6c, brown open symbols) compared to exosomes isolated with NT80 + 82 (Fig. 6b, brown closed symbols). As shown previously, exosome production increased daily (1–5 days) with ex vivo culture of HAM/TSP PMBCs with a corresponding increase in Tax detection from isolated exosomes (Figs. 3e, 4a). This increase in Tax^+^ exosomes additionally correlated with increased CTL lysis (Fig. 6c, NT day 1, day 3, day 5). Specifically, samples that were nanotrapped with NT80 + 82 particles (NT) at day 1 (brown closed circle) yielded 10%, while NT day 3 yielded 20% specific lysis, and NT day 5 yielded 30% specific lysis of targets at 20:1 and decreased at lower E:T ratios. Samples that were nanotrapped with control NT86 particles do not capture exosomes (ctrl) at day 1, day 3, and day 5 and were therefore unable to sensitize targets for lysis at any E:T ratio after pulsing of targets (Fig. 6c, brown open circle). Further characterization of the Tax^+^ exosomal sensitization of targets for CTL lysis is shown in Fig. 6d, which demonstrated a dose-dependent response of sensitization with increasing concentrations (250 μL to 1 mL) of Tax^+^ exosomes. Nanotrapping from the standard 1 mL (green closed circle) of tissue culture supernatant yielded ~ 18% specific lysis, while nanotrapping from 500 μL (green closed square) yielded 15% specific lysis of targets and exosomes isolated from 250 μL (green closed triangle) of starting tissue culture supernatant yielded 10% specific lysis of targets at an E:T of 20:1. Each sample concentration showed decreased specific lysis as lower E:T ratios. While most HAM/TSP PBMC derived exosomes were able to sensitize targets for CTL response, the magnitude of this response varied among patients (Fig. 6e). CTL assay using HAM/TSP patient PBMC-derived exosomes showed that exosomes from 2/11 HAM/TSP patients did not sensitize targets for specific lysis at any E:T ratio tested (Fig. 6e, representative of 8 HAM/TSP patients). Collectively, these results demonstrate that exosomes containing HTLV-1 Tax can contribute to a functional immune response by sensitizing target cells to specific lysis by antigen-specific CTLs.

## Discussion

There is significant interest in the role of exosomes in viral diseases and as a potential biomarker in these disorders. Many viruses enter cells through the endosomal compartment and coopt proteins involved in endosome/exosome formation and shuttling, using them for viral particle egress [23]. Indeed, herpesviruses, flaviviruses, bunyaviruses, and lentiviruses incorporate viral messages into exosome cargo [23]. It has been suggested that these released exosomes may dysregulate the immune response [67, 68]. Since the observation that HTLV-1 Tax could be found in exosomes produced by HTLV-1 infected cell lines in vitro [47], it became important to characterize exosomes ex vivo from patients with HTLV-1 associated neurologic disease. HAM/TSP, a neurologic disorder associated with higher levels of HTLV-1 proviral loads [69], is characterized by a widespread inflammatory immune response and decreased immune suppression [5, 70].

Here we show that PBMCs from HAM/TSP patients produced exosomes containing HTLV-1 Tax protein (Fig. 3) in ex vivo cultures, consistent with reports demonstrating Tax in exosomes produced by HTLV-1 infected cell lines [47]. HTLV-1 Tax is a pleiotropic transactivating protein known to be critical in immune activation detected in HAM/TSP [71]. It is also highly immunogenic, which results in a strong Tax-specific immune response [72–74]. HTLV-I Tax-specific CTLs have been shown to be elevated in peripheral blood and even higher in CSF of HAM/TSP patients, in part driven by high levels of HTLV-I proviral loads demonstrated in these compartments [18, 62, 75]. These observations have supported the hypothesis that these antigen-specific responses are associated with the neuropathogenesis of HAM/TSP, and therefore may be reasonable targets for immune interventions that either directly target the effector CTL or the antigen that stimulates this response. Activated T cells are a hallmark of HAM/TSP, but within the CNS there is a paucity of HTLV-1 infected cells, and even if present, have been postulated to be nonproductively infected [9].

**Fig. 7:**
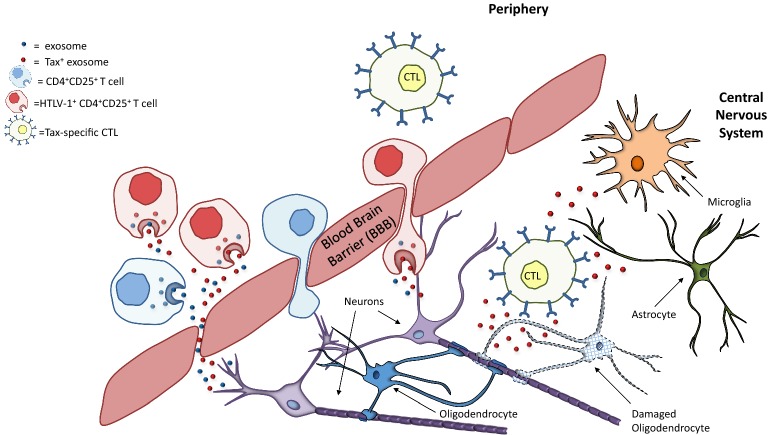
Hypothesized contribution of exosomes to HAM/TSP immunopathogenesis. HTLV-1 infected PBMCs, especially HTLV-1 infected CD4^+^CD25^+^ T cells, produce exosomes containing HTLV-1 Tax. These cells are present in the periphery and produce Tax^+^ exosomes in the periphery that can potentially cross the blood brain barrier (BBB). However, these infected T cells are also capable of crossing the BBB and producing exosomes in the CNS. Both scenarios may explain the presence of Tax^+^ exosomes in HAM/TSP patient CSF. These Tax^+^ exosomes can then be taken up by CNS resident cells, including microglia, oligodendrocytes, astrocytes, etc. and become targets for lysis by HTLV-1 Tax-specific CTLs which cross the BBB. The resulting destruction perpetuates the neuroinflammation observed in HAM/TSP

While HTLV-1 proviral DNA levels are significantly increased in T cells in the CSF of HAM/TSP patients compared to peripheral blood [63, 76], no cell-free virus can be demonstrated in CSF supernatant [61, 77]. This is not unexpected since HTLV-1 is known to be highly cell-associated [78]. However, we could demonstrate exosomes containing HTLV-1 Tax in HAM/TSP CSF supernatant (Fig. 5). The presence of Tax in exosomes in CSF supernatants suggests that exosomes either cross the blood–brain barrier (BBB) after production by infected cells in the periphery, or by infiltrating infected T cells within the CSF (schematic representation Fig. 7). While monocytes and macrophages comprise a significant portion of exosome production in the periphery [79, 80], the vast majority of immune cells infected in HAM/TSP are activated T cells [5], making them a likely source of the Tax^+^ exosomes found in our samples. Indeed, HAM/TSP CD4^+^CD25^+^ subset produces significantly more exosomes than HAM/TSP CD4^+^CD25^−^ T cells (Fig. 3h) and CD8^+^CD25^+^ T cells. The CD4^+^CD25^+^ subset also includes regulatory T cells (Tregs), a subset known to be dysfunctional in HAM/TSP [5], and has been reported to be a predominant source of exosomes in mice [34].

While Tax^+^ exosome detection was apparent in HAM/TSP CSF (12/20), we could not find evidence of Tax^+^ exosomes in a small group of asymptomatic carrier (AC) CSF samples (data not shown) (and, as expected, there were none detected from HTLV-1 negative patients (0/5, Fig. 5b, c). Of interest, we were able to detect Tax^+^ exosomes in the CSF sample of an individual initially classified as an AC who began developing clinical signs and symptoms of HAM/TSP on the date the CSF was obtained (unpublished observation). Collectively, these observations suggest that the detection of Tax in exosomes isolated from the CSF could serve as an additional biomarker of progression to HAM/TSP. The lack of Tax protein present in the exosomes from all HAM/TSP CSF samples probably reflects the diversity in the HAM/TSP patient population and the biology of the disease.

In HAM/TSP, HTLV-1 Tax in exosomes may also serve as a source of antigen in the disease. As shown in this present study, HAM/TSP PBMCs produced exosomes containing Tax in culture (Fig. 4), and these ex vivo Tax-containing exosomes were able to sensitize target cells for Tax-specific CTL lysis (Fig. 6). Exosomes containing viral proteins may therefore be an underappreciated source of antigen that drives continued immune activation in HAM/TSP. Histologically, antigen-specific CTLs have been demonstrated in the CNS and leptomeninges in HAM/TSP patients, suggesting that this disease is immunopathologically mediated [17, 64]. Our observation that Tax^+^ exosomes can be detected in the CSF of HAM/TSP patients suggests exosomal transference of HTLV-1 antigens to antigen presenting cells (APCs) may also occur in the CNS, and could play a role in perpetuating neuroinflammation in HAM/TSP through damage to various CNS resident cells such as astrocytes, microglia or oligodendrocytes (Fig. 7). Sensitization of CTLs by Tax^+^ exosomes suggests a potential role in HAM/TSP disease initiation and/or progression. Future experiments targeting diminution of Tax content in exosomes through knockdown or silencing methods will be of value since recently it has been shown that Tax expression is essential for HAM/TSP PBMC survival [81].

The observations in this study on the detection of HTLV-1 viral antigens in exosomes found in virus-free CSF supernatant has implications for other virally associated neuroinflammatory diseases. For example, Ebola virus has been shown to be associated with long-term neurologic sequelae even though the virus is thought to have been cleared [81, 82], and while Ebola viral proteins have been detected within exosomes in vitro [83], this has not yet been shown to be the case within patient CSF material. As we have demonstrated that HAM/TSP CSF supernatant, which is HTLV-1 negative, contains HTLV-1 Tax^+^ exosomes, exosomes containing viral antigens could be a potential biomarker for neurologic disorders associated with viral infections in which the virus may be absent in CSF. Indeed, recent data has demonstrated that exosomes containing HIV antigens are still present in the serum of patients under highly active antiretroviral therapy (HAART) for whom there is no detectable virus [53], indicating that tracking of exosomal cargo could monitor viral reservoirs. Furthermore, exosomes could be a tool for pathogen discovery in a wide variety of undiagnosed aseptic meningitides and encephalitides for which a virus is suspected but undetectable [84, 85]. The etiology for over 50% of meningitis and encephalitis cases is currently unknown [86, 87]. Collectively, the observations presented in this report on exosomes containing viral antigens in the CSF of patients with HTLV-1 associated neurologic disease will be of relevance to many neuroinflammatory diseases in which viruses are thought to play a role.

## Conclusions

The understanding of exosomes and their contribution to human disease is a quickly expanding field. Evidence suggests that exosomes play a large role in immunity and infection. Some viruses have been shown to alter exosome cargo with viral components in another mechanism of immune evasion. Here we show that HTLV-1 virus incorporates HTLV-1 Tax, an important viral regulatory protein, into exosomes found in the CSF and produced by HTLV-1 infected PBMCs. These exosomes could sensitize cells for lysis by HTLV-1 specific CTLs which are indicated in HAM/TSP neuropathology. This suggests that exosomes may have a functional role in CNS damage in HAM/TSP patients.

## Methods

### Cell lines

HTLV-1 infected cell lines HUT102, MT-2 and C8166 and the uninfected Jurkat cell line were maintained in exosome-free (Exo-free) complete RPMI. Specifically, FBS was spun at > 90,000×*g* for 2 h and filtered through a 0.22 μm MCE filter (EMD Millipore, Darmstadt, Germany), after which it was considered exosome-free. Exo-free CRPMI consisted of RPMI 1640 supplemented with 10% Exo-free FBS, 100 U/mL penicillin, 100 μg/mL streptomycin sulfate, and 2 mM l-glutamine.

### Patient samples

A total of 36 HAM/TSP, 10 ND, and 2 HTLV-II patient PBMC samples were used for ex vivo incubation and later exosome isolation. PBMCs were isolated by Ficoll-Hypaque (Lonza, Walkersville, MD) centrifugation, and were cryopreserved in liquid nitrogen prior to use. All NDs were noted to be healthy and HTLV-1 negative. Briefly, PBMCs were placed in Exo-free CRPMI at 8 × 10^6^ cells per well in a 12-well plate and incubated at 37 °C for 5 days. ND PBMCs were maintained in culture with the addition of 100 IU/mL recombinant human (rh) IL-2. Culture supernatant was collected and then spun at 1300 rpm for 10 min to remove cells. Spun supernatant was then pushed through a 0.22 μm MCE filter (EMD Millipore) to remove large debris, apoptotic bodies and extracellular vesicles > 220 nm. Cerebrospinal fluid (CSF) was obtained through lumbar puncture of study participants by neurologist and nurse practitioners of the Neuroimmunology Clinal group. CSF was spun at 1300 rpm for 10 min to remove cells and supernatant was stored in 1 mL aliquots at − 80 °C prior to use.

### Exosome isolation

Nanotrap^®^ particles NT80 (Ceres #CN1030) and NT82 (Ceres #CN2010) were provided by Ceres Nanosciences, Inc. (Manassas, VA) and have previously been shown to concentrate exosomes from media [47]. Each was mixed into PBS (w/o Ca^+^ or Mg^+^) for a final 30% slurry (30 μL each NT in a total 100 μL volume with PBS). Thirty microliters of 30% NT slurry was then added to 1 mL of ex vivo PBMC supernatant and rotated overnight at 4 °C or at room temperature for 1 h. After trapping, samples were spun at 13,000 rpm for 5 min and then washed with 500 μL DEPC H_2_0. Exosome control NT86 (ctrl NT) was similarly placed in PBS (w/o Ca^+^ or Mg^+^) for a final 30% slurry. When working with CSF, samples were first diluted 1:1 in PBS (w/o Ca^+^ or Mg^+^) prior to nanotrapping.

### Western blotting

HTLV-1 Tax, Alix, CD63, and Actin were detected from nanotrapped exosomes by Western blot. Briefly, after washing, samples were resuspended in 100 μM Tris–HCl, 8 μM urea, 1% triton. After the addition of 4× LDS and DTT, samples were heated at 95 °C for 6 min, with a vigorous vortex for 5–10 s every 2 min. After heating, samples were run on a 4–12% Novex Bis–Tris 1.0 mm gel (ThermoFisher Scientific, Waltham, MA) and then transferred in an X-blot module onto a 0.45 μm nitrocellulose membrane. After blocking with 3% BSA in TBS, membranes were probed with anti-Tax (Lt4 1:100, mIgG3; Kindly provided by Dr. Yuetsu Tanaka), anti-CD63 or anti-Alix (1:100; Santa Cruz Biotech, Dallas, TX) overnight at 4 °C. For imaging, anti-ms HRP secondary antibody (Jackson ImmunoResearch, Westgrove, PA) was applied at 1:5000 or goat anti-ms IR 680RD secondary (1:10,000. LI-COR Biosciences, Lincoln, NE) was used for CSF samples. For detection of Actin, blots were stripped for 5–10 min with Restore PLUS Western Blot Stripping Buffer (ThemoFisher Scientific), reblocked and reprobed with Actin (Santa Cruz Biotech) at 1:100 overnight at 4 °C.

### Acetylcholinesterase enzyme (AchE) activity assay

AchE activity of exosomes after nanotrapping was carried out as described in detail previously [55] with brief modification. Amplex^®^ Acetylcholine/Acetylcholine Esterase Activity Assay Kit (Thermo; A12217) was used following the manufacturer’s instructions. Briefly, a 1× running buffer negative control (20 mL of H_2_O and 5 mL of 5× reaction buffer) and two positive controls, one consisting of acetylcholinesterase and one consisting of hydrogen peroxide, were made and plated on a 96-well plate. Exosomes were treated and fluorescence of acetylcholinesterase activity was measured with a GLOMAX multidetection system (Promega, Madison, WI) every 15 min for 1 h to find optimal activity. Changes in absorption were monitored at 412 nm during incubation period at 37 °C [88].

### Electrochemical ELISA

For CD81 and HTLV-1 Tax, nanotrapped exosomes were resuspended in 1× TBS for assessment by MESO Enzyme Linked Immunosorbent Assay (ELISA) (MSD Technologies, Rockville, MD). Briefly, samples were freeze-thawed for 4 cycles of 5 min each and then blotted onto a MESO Sector Imager 96-well high-bind plate (MSD Technologies) in duplicate wells overnight. The plate was then blocked with blocker A for 2 h, washed with PBS ×2 and then probed with anti-CD81 (Santa Cruz Biotech) or anti-HTLV-1 Tax (Lt4) for 1 h, shaking. After a repeat wash, the plate was probed with goat anti-ms sulfa secondary Ab (1:100, MSD Technologies). After a final wash, MSD Read buffer was added at 1:2 dilution in Ultra Pure Water and the plate read using an MSD Sector Imager (courtesy of Dr. Avi Nath).

### ^3^H-thymidine assay for lymphocyte proliferation

Thawed ND or HAM/TSP PBMCs were placed on 96-well plate in triplicate at 3 × 10^5^ cells/well in 10% Exo-Free CRPMI and allowed to proliferate for 3–5 days in culture at 37 °C. At 3, 4, or 5 days, plates were removed from culture and 50 μL of tritium solution was added to each well. Cells were incubated for 4 h at 37 °C after which they were washed and harvested onto a film mat. Five milliliters of scintillation fluid was added after which thymidine incorporation was measured using a β plate counter (Microbeta TriLux 1450, Perkin Elmer, Waltham MA).

### T cell isolation

To understand which T cell subsets were responsible for exosomes found in PBMC culture, sorting was performed on thawed or previously activated ND PBMCs. Activation occurred with addition of 100 ng/mL anti-CD3 (HIT3a, eBiosciences) and 300 IU/mL rhIL-2 24 h prior to sorting. At least 5 × 10^7^ PBMCs were stained in FACS buffer (1% FBS, 0.1% Sodium Azide, PBS) with 10 μL/1 × 10^7^ cells of anti-CD4 APC, anti-CD8 FITC and anti-CD25 PE (all from BD Biosciences). Cells were then sorted into CD4^+^CD25^−^, CD4^+^CD25^+^, CD8^+^CD25^−^, CD8^+^CD25^+^ T cell subsets on a BD FACS Aria flow cytometer. After sorting, cells were briefly placed in 20% FBS, 2× antimycobacterial, antibacterial (ThermoFisher Scientific) RPMI 2 mM l-glutamine. Cells were then spun at 1300 rpm for 10 min and resuspended in Exo-free CRPMI supplemented with 100 IU/mL hrIL-2 and plated at 7.5 × 10^5^ cells per well in a 12-well plate for 5 days at 37 °C. For HAM/TSP PBMCs, no exogenous stimulation was provided. Endogenous activation of HAM/TSP PBMC was achieved by incubation of PBMCs for 18–24 h prior to FACS sorting as described above.

### CTL assay

HTLV-1 Tax specific CTL line and target B cell line were generated from an HLA A0201 HAM/TSP patient and cryopreserved prior to use. As described previously [64], CTLs were primed with Tax peptide 11-19 weekly for expansion and maintenance in CTLA media (IMDM with 5% FBS, 100 μg/mL penicillin, 100 μg/mL streptomycin sulfate, 12.5 mM sulfinpyrazone, 2 mM l-glutamine) for up to 1 month. Clones were tested for efficiency of target killing prior to use. Targets B cells were pulsed for 1 h at 37 °C with Tax peptide 11-19 or nanotrapped exosomes from tissue culture supernatant of 11 separate HAM/TSP PBMC ex vivo cultures and 4 ND PBMC cultures. One HAM/TSP supernatant was nanotrapped with NT86 as a negative control. Anti-HLA Class I ab (MCA81EL, Serotec (BioRad), Oxford UK) was added to CTLs after co-culture with targets at 5 μg/mL. The CTL Europium cytotoxity release assay was performed as outlined in the DELFIA protocol (Perkin Elmer Inc., Waltham, MA). Specific lysis was calculated as a percentage:

$${{\left( {{\text{sample x}}\, - \,{\text{Target}}_{\text{only}} } \right)} \mathord{\left/ {\vphantom {{\left( {{\text{sample x}}\, - \,{\text{Target}}_{\text{only}} } \right)} {\left( {{\text{Target}}_{ \text{max} } \, - \,{\text{Target}}_{\text{only}} } \right)}}} \right. \kern-0pt} {\left( {{\text{Target}}_{ \text{max} } \, - \,{\text{Target}}_{\text{only}} } \right)}}\, \times \, 100\% .$$

### Exosome characterization by nanosight and nanoFACS

Tissue culture supernatant or resuspended nanotrapped exosomes were pushed through a syringe at a total volume of 300 μL into the chamber of the NanoSight LM10 instrument (Malvern Instruments, Worcestershire, UK) for extracellular vesicle visualization and quantification. Briefly, a laser beam is passed through the chamber containing the sample and light scattering and Brownian motion is quantified as a vesicle size distribution and quantity. For exosome visualization by flow cytometry (nanoFACS), samples were loaded into a Beckman Coulter MoFlo XDP cell sorter (Beckman Coulter, Brea, CA) as described extensively by Danielson et al. [56].

### Statistical analysis

Bar graphs and scatter plots were generated in GraphPad Prism 7.0 (GraphPad, San Diego, CA). Two-tailed unpaired *t*-tests were run to establish difference between groups and significance was considered anything with a *p*-value < 0.05. The two-tailed Fisher exact test was used to assess differences between groups on parametric questions (Tax^+^ or Tax^−^ exosomes) using GraphPad QuickCalcs software. Statistical analyses of 2 × 2 contingency tables were performed using GraphPad QuickCalcs software with two-tailed Fisher exact tests. *p*-values < 0.05 were considered significant.

## Additional file

**Additional file 1: Figure S1.** Characterization of Nanotrapped (NT80 + 82) exosomes by Nanosight. Tissue culture supernatants from HTLV-1 infected cells prior to nanotrapping (Pre-NT) and after nanotrapping (+NT) were analyzed for size and concentration by Nanosight. Nanotrapped exosomes (+NT) were resuspended in 300 μL for imaging. NT80 + 82 particles alone (NT Alone) were also analyzed. Pre-nanotrapped (Pre-NT) and post-nanotrapped (post NT) vesicle distributions were likewise analyzed according to size and shown in the right panel inset.

## Abbreviations

Ab: antibody
AC: asymptomatic carrier
AchE: acetylcholinesterase enzyme
Ag: antigen
APC: antigen presenting cell
BBB: blood brain barrier
C81: C8166 cell line
CNS: central nervous system
CSF: cerebrospinal fluid
CTL: cytotoxic T lymphocyte
EBV: Epstein Barr virus
ELISA: enzyme linked immunosorbent assay
ESCRT: endosomal sorting complexes required for transport
HAART: highly active anti-retroviral therapy
HAM/TSP HTLV-1: associated myelopathy/tropical spastic paraparesis
HT: HUT102 cell line
HTLV-1: human T cell lymphotropic virus type 1
ILV: intraluminal vesicles
Ju: Jurkat cell line
MS: multiple sclerosis
MVB: multivesicular body
ND: normal donor
NT: nanotrap particles
PBMC: peripheral blood mononuclear cell
Treg: regulatory T cell
