# Supplementary figures and images for: Viral antigens detectable in CSF exosomes from patients with retrovirus associated neurologic disease: functional role of exosomes
Source: Clin Transl Med. 2018 Aug 27;7:24. doi: 10.1186/s40169-018-0204-7 (PMC6110307; doi:10.1186/s40169-018-0204-7)

## Slide 1
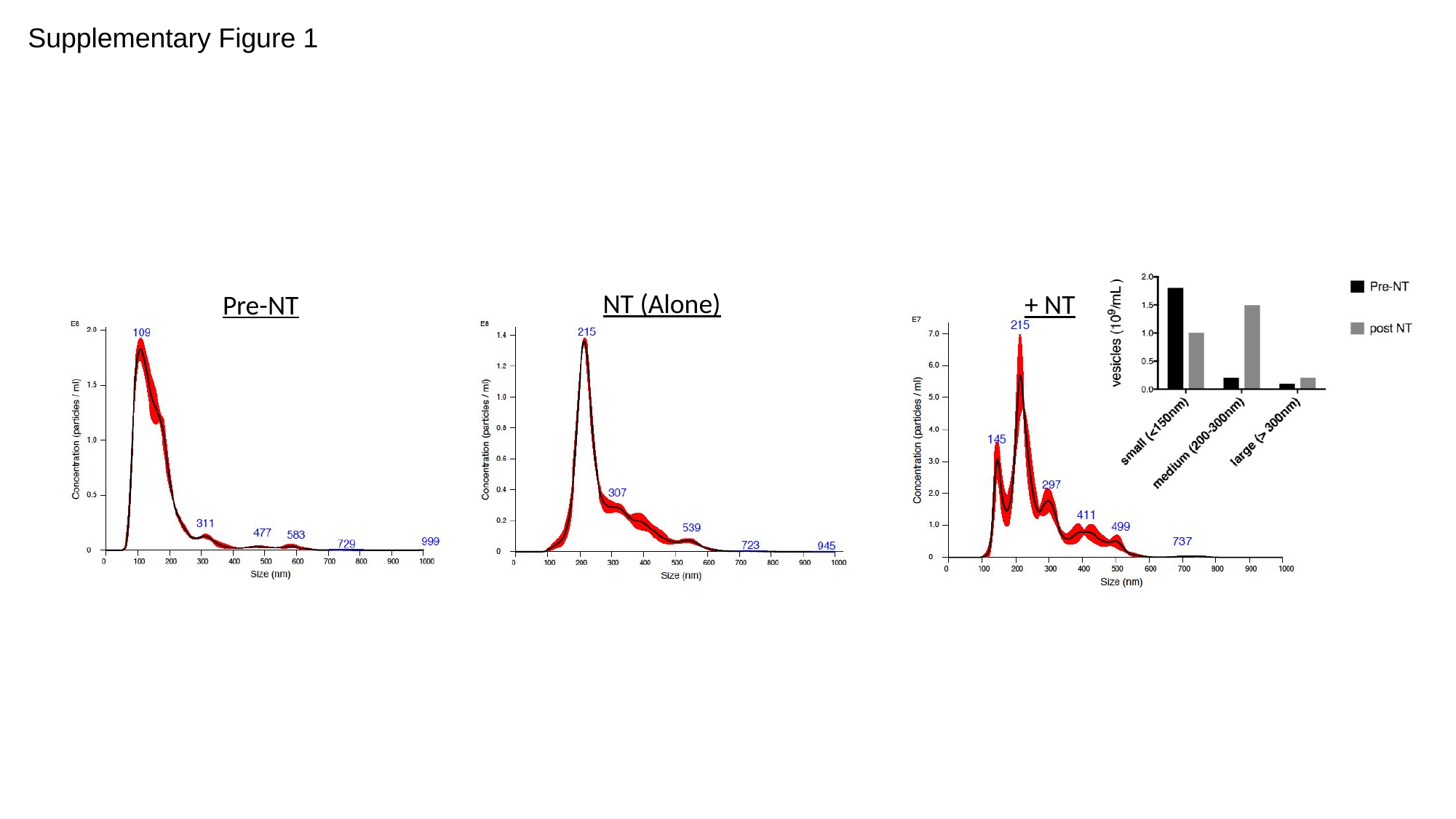

Supplementary Figure 1
NT (Alone)
+ NT
Pre-NT

Supplement: Supplementary file 1 — Additional file 1: Figure S1. Characterization of Nanotrapped (NT80 + 82) exosomes by Nanosight. Tissue culture supernatants from HTLV-1 infected cells prior to nanotrapping (Pre-NT) and after nanotrapping (+NT) were analyzed for size and concentration by Nanosight. Nanotrapped exosomes (+NT) were resuspended in 300 μL for imaging. NT80 + 82 particles alone (NT Alone) were also analyzed. Pre-nanotrapped (Pre-NT) and post-nanotrapped (post NT) vesicle distributions were likewise analyzed according to size and shown in the right panel inset. [file 40169_2018_204_MOESM1_ESM.pptx]
